# Tea plant–legume intercropping simultaneously improves soil fertility and tea quality by changing *Bacillus* species composition

**DOI:** 10.1093/hr/uhac046

**Published:** 2022-02-19

**Authors:** Zhi Huang, Chunhong Cui, Yajun Cao, Jinghui Dai, Xiaoyue Cheng, Shaowei Hua, Wentao Wang, Yu Duan, Evangelos Petropoulos, Hui Wang, Lixiang Zhou, Wanping Fang, Zengtao Zhong

**Affiliations:** 1 College of Life Sciences, Nanjing Agricultural University, Nanjing 210095, China; 2 College of Resource and Environment Sciences, Nanjing Agricultural University, Nanjing 210095, China; 3 College of Horticulture, Nanjing Agricultural University, Nanjing 210095, China; 4 School of Engineering, Newcastle University, Newcastle upon Tyne, NE1 7RU, UK

## Abstract

The tea plant is an economically important crop in China, but long-term monoculture and substantial chemical nitrogen fertilizer input cause soil acidification, which in turn affects the nutrient supply and tea quality. Intercropping has drawn more attention in tea gardens because this pattern is expected to improve soil fertility and tea quality and change the soil microbial community composition. However, the roles of some key microorganisms in rhizosphere soils have not been well characterized. Here, a “soybean in summer and smooth vetch in winter” strategy was used in a tea garden to investigate the effects of intercropped legumes on soil fertility, tea quality, and potential changes in beneficial bacteria such as *Bacillus*. Our data showed that when soybeans were turned into the soil, the intercropping system exhibited higher soil organic matter (SOM), total nitrogen (TN), tea quality indices, and expression of the *Camellia sinensis* glutamine synthetase gene (*CsGS*). Notably, intercropping significantly affected the bacterial communities, decreasing the relative abundance of *Bacillus* but increasing its absolute abundance. *Bacillus amyloliquefaciens* BM1 was isolated from intercropped soil and showed outstanding plant growth-promoting (PGP) properties when co-inoculated with rhizobia. In winter, intercropping with smooth vetch had a beneficial effect on soil properties and tea quality. Co-inoculation with strain BM1 and *Rhizobium leguminosarum* Vic5 on smooth vetch (*Vicia villosa*) produced huge improvements in SOM, TN, and tea leaf quality, which were accompanied by the highest level of amino acids and the lowest levels of polyphenols and caffeine (*p* < 0.05). Our findings demonstrate that intercropping with some legumes in the tea garden is a strategy that increases SOM, TN, and tea quality, and the optional use of some PGP *Bacillus* species produces an amplification effect.

## Introduction

The tea plant (*Camellia sinensis* L.) is one of the most economically important crops in many developing countries, including China, India, Kenya, and Sri Lanka. In China, there were approximately 3.10 million ha of tea planting area and 2.78 million tons of total yield in 2019. Nitrogen (N) is particularly important for tea plants because it is associated with their growth and with the biosynthesis of amino acids that affect the quality properties of tea [1]. After the uptake of active N, the *C. sinensis* glutamine synthetase gene (*CsGS*) and glutamate synthase gene (*CsGOGAT*) play vital roles in transferring N to amino acids, particularly theanine [2]. Therefore, to maintain high tea yield and quality, chemical fertilizers, particularly N fertilizers, have been widely used. In some areas, the average use of N has reached up to 521 kg/ha per year [3], an amount that exceeds the upper limit of N fertilizer (450 kg/ha) [4]. There is no question about the positive effects of chemical fertilizer on increasing tea yield, but prolonged excessive application of fertilizers often leads to environmental risks, such as soil degradation and acidification, nutrient waste, water body eutrophication, and declining tea quality [5–7]. As a result, these potential problems may reduce the economic value and market competitiveness of the produced tea. Currently, considerable work has been performed on the reduction of chemical fertilizers in tea gardens, as well as the effects on tea yield and quality of low-cost and eco-friendly methods such as the use of habitat management [7].

Intercropping is a useful agricultural strategy in which one plant species is grown alongside two or more crops in the same area at the same time, thereby increasing crop quality and yield. Compared with monoculture, intercropping improves soil fertility and plant diversity, reduces weeds, and improves light interception and utilization [8, 9]. Tea plants are perennial shrubs, and intercropping with other plants such as chestnut and legumes has been developed in a wide range of forms and applied in different tea gardens [10, 11]. In some intercropping systems, intercropped plants may compete for soil N, and the yield and/or tea quality may be adversely affected. For example, Ma et al. [12] found that in chestnut-tea intercropping patterns, compared with monoculture, intercropping improved the growth and quality of tea plants (including the theanine content in tea leaves) but also decreased total amino acids and catechin contents. Therefore, it is particularly important to select appropriate intercropping plants for tea. Compared with other plants, legumes can obtain N from the atmosphere through biological nitrogen fixation (BNF) when they form symbiotic relationships with N_2_-fixing bacteria, particularly in low-input agricultural systems [9].

Soil microorganisms play a vital role in agricultural ecosystems, as they are involved in a number of soil functions and ecological services. The soil in most tea gardens is acidic, both because of the tea plant itself and because of the application of N fertilization [6]. Previous studies have shown that long-term tea monoculture often has a negative effect on soil microbial diversity and beneficial microbes [13, 14], as well as soil enzymatic activity [5]. For example, Li et al. [15] studied the bacterial community composition of soils subjected to continuous (10- and 20-year) tea orchards and found that the relative abundance of some beneficial bacteria, such as *Pseudomonas* and *Bradyrhizobium*, decreased over time. By contrast, plant species and intercropping patterns can greatly affect soil properties and microbial community composition. For example, Shen and Lin [16] investigated the short-term effects of soybean intercropping in a tea garden, showing that the intercropping pattern not only increased soil EC and available P, K, and some other microelements but also increased the relative abundances of Acidobacteriaceae, Burkholderiaceae, Rhodanobacteraceae, and Sphingomonadaceae, which are considered to be organic matter decomposers and/or plant growth-promoting bacteria.

Specifically, in agricultural systems, *Bacillus*, *Azobacter*, and *Pseudomonas* are routinely defined as plant growth-promoting bacteria (PGPB) and directly and/or indirectly contribute to crop productivity [17, 18]. In tea gardens, Arafat et al. [19] found that *Bacillus* and other beneficial bacteria were significantly reduced in long-term tea plantations. However, there is still insufficient information on the actual effects of tea garden intercropping systems on the abundance of *Bacillus*. Previous studies have confirmed that co-inoculation of some *Bacillus* and rhizobia species has a synergistic effect on legume growth, producing higher biomass, nodule numbers, and levels of nitrogenase compared with the inoculation of rhizobia alone [20, 21]. To date, we still have only a limited understanding of the effects of *Bacillus* and rhizobia co-inoculation on legumes in tea gardens. Microbial community composition can now be conveniently analyzed using high-throughput sequencing technologies. Predominant species in communities are often defined based on relative abundance, but it remains difficult to understand the dynamics of species across multiple samples with this approach [22, 23]. Recently, some microbial quantification techniques, such as flow cytometry and qPCR, have been used to obtain the absolute abundance of the microbial community in various environments [24, 25]. Thus, combining methods based on high-throughput sequencing and qPCR will provide further insight into the microbial community in diverse environments.

**Figure 1:**
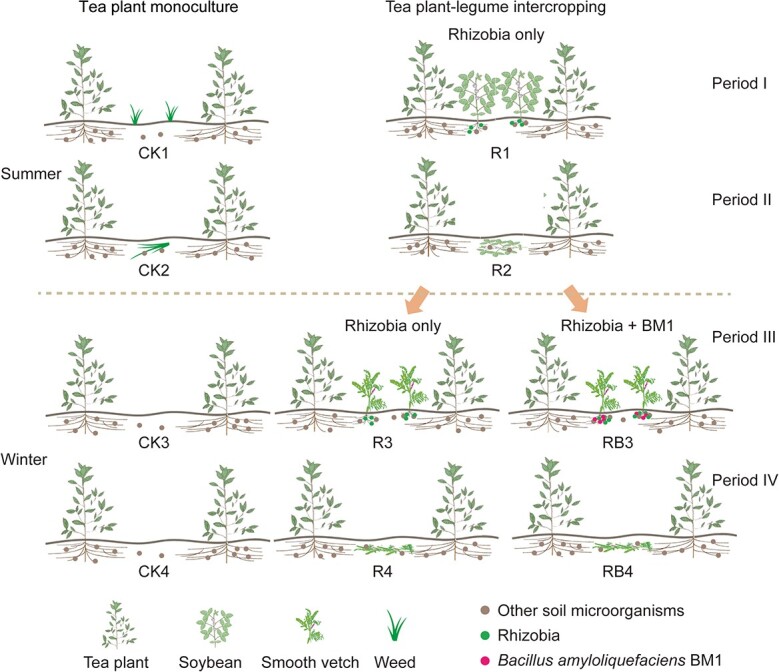
Conceptual model of the experimental design in this study. In summer, soybean was used as the intercropping plant, and *Br. diazoefficiens* USDA110 was inoculated as rhizobia (R) that formed nodules on soybean. Period I indicates that the soybeans were flowering and/or podding, and period II indicates that the soybeans were turned into the soil for approximately 40 days. Monoculture was used as the control (CK). In winter, smooth vetch and *R. leguminosarum* Vic5 were used as the intercropped plant and related rhizobia, respectively. In addition to monoculture and single inoculation, co-inoculation with rhizobia and *B. amyloliquefaciens* BM1 was also assessed under field conditions (RB). Period III indicates that smooth vetch was flowering and/or podding, and period IV indicates that smooth vetch was turned into the soil for approximately 30 days.

In this study, the effects of intercropping tea plants with different types of legumes (soybean, mung bean, and smooth vetch) were examined in field experiments. We hypothesized that tea plant–legume intercropping systems would help to improve soil fertility and tea quality and that this pattern would change the soil microbial community composition and increase the counts of beneficial microorganisms such as *Bacillus*. Therefore, the first objective was to estimate the effects of intercropping on soil fertility and tea plant performance. Second, we aimed to screen some plant growth-promoting (PGP) *Bacillus* species and assess their PGP effects on intercropped legumes under both pot and field conditions. Specifically, the experimental trial was performed in field conditions, and a conceptual model of the experimental design was prepared (shown in Fig. 1). In this study, the overall objective was to provide more information for understanding intercropping in tea gardens.

## Results

### The growth performance of tea plant–soybean intercropping

**Figure 2:**
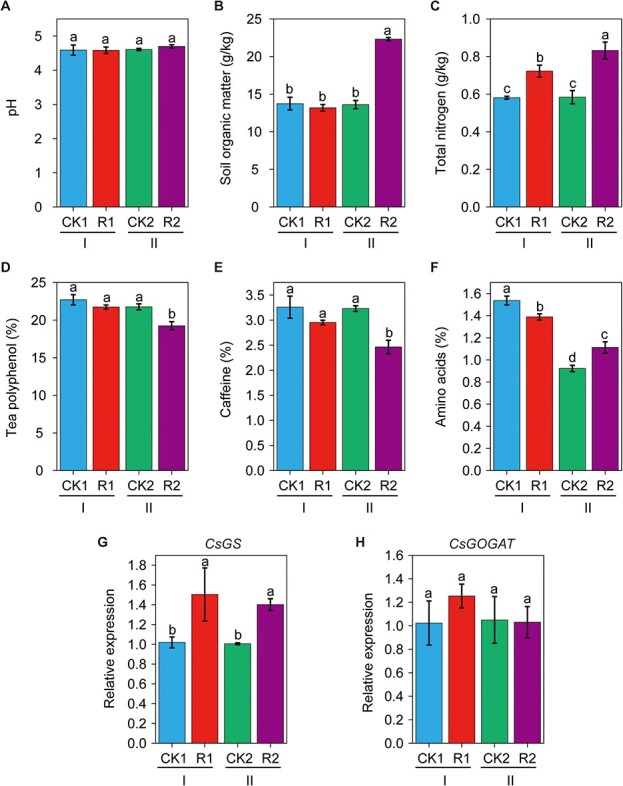
The effects of monoculture and intercropping (soybean–tea plant) under field conditions. **a** Soil pH. **b** Soil organic matter. **c** Total nitrogen. **d** Tea polyphenols in tea leaves. **e** Caffeine in tea leaves. **f** Amino acids in tea leaves. **g** Relative expression of *CsGS*. **h** Relative expression of *CsGOGAT*. I and II represent periods I and II, respectively. CK1 and CK2, tea plant monoculture in periods I and II; R1 and R2, intercropping with soybean in the tea garden in periods I and II. All data are shown as the mean ± SD (n = 3). Different letters represent significant differences (*p* < 0.05).

In the tea garden, both soybean and mung bean could adapt to the acidic conditions and exhibited adequate growth (Supplementary Fig. 1). As shown in Fig. 2a, intercropping with soybeans had no effect on soil pH, regardless of the experimental period. Compared with CK1, R1 did not show an increase in the content of SOM (Fig. 2b, *p* > 0.05) but did increase soil total N (TN) (Fig. 2c, *p* < 0.05) in period I. In period II, SOM and TN were higher in intercropping than in monoculture by 63.9% and 43.1%, respectively (Fig. 2b and 2c, *p* < 0.05). In period I, no significant difference in polyphenol and caffeine levels was observed between intercropping and monoculture (Fig. 2d and 2e), and a decrease in amino acid content was observed for R1 compared with CK1 (Fig. 2f, *p* < 0.05). As expected, in period II, tea polyphenol and caffeine contents were significantly lower for intercropping than for monoculture by 11.6% and 23.8%, respectively (Fig. 2d and 2e, *p* < 0.05), and the content of amino acids in leaves was enhanced by 20.7% for intercropping (Fig. 2f, *p* < 0.05). To examine the expression of genes related to the synthesis of amino acids in tea leaves, *CsGS* and *CsGOGAT* were analyzed by qPCR using gene-specific primers. The relative expression of *CsGS* showed a significant increase in periods I and II between intercropping and monoculture (Fig. 2g). By contrast, there was no significant difference in the expression of *CsGOGAT* between these periods (Fig. 2h). Intercropping with mung bean (*Vigna radiata*) in the tea garden also showed similar patterns with regard to soil properties, tea quality indices, and the expression of *CsGS* and *CsGOGAT* (Supplementary Fig. 2).

### Bacterial diversity and community composition

A total of 826 359 high-quality sequences were obtained after sequencing and quality control. There were 33 010–59 542 valid reads obtained per sample. Overall, 5645 operational taxonomic units (OTUs) were found in all soil samples. The corresponding rarefaction curves tended to nearly saturate at the selected sequencing depth (not shown). Bacterial alpha diversity indices are shown in Supplementary Table 1, suggesting that none of the soil samples differed significantly in either the OTU number or the Shannon or Chao 1 indices. The beta diversity was calculated according to the Bray–Curtis method at the OTU level. As shown in Supplementary Fig. 3, the soil samples were separated from each other. Permutational multivariate analysis of variance (PERMANOVA) indicated that intercropping with soybean in the tea garden significantly changed the soil bacterial community (*p* < 0.05).

**Figure 3:**
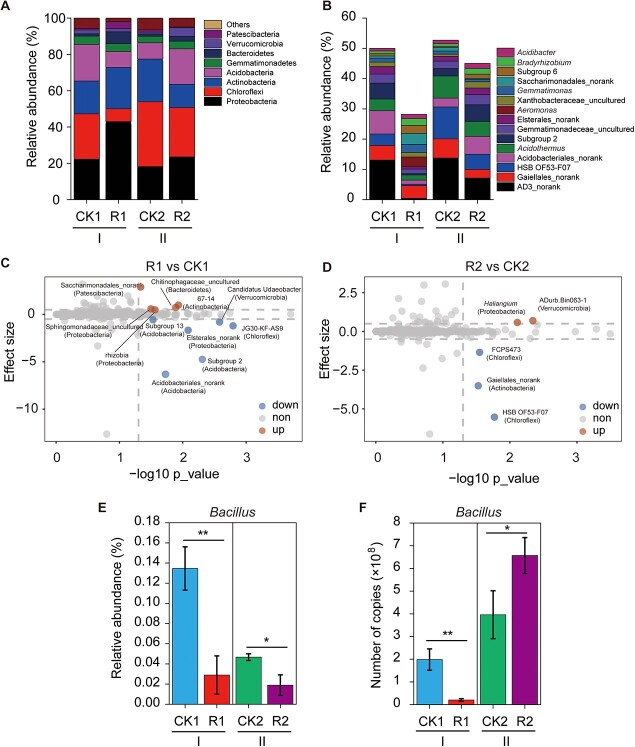
Intercropping changed the soil bacterial community composition in the tea garden. **a** Relative abundance of major bacterial communities (>1%). **b** Top 15 genera of each soil sample. **c** and **d** Differences in bacterial groups at the genus level between R1 and CK1 and R2 and CK2, respectively. The data were analyzed using STAMP with two-group comparisons. The effect size represents the difference in the relative abundance of some bacterial genera between two samples. The red and blue dots indicate samples with *p* values <0.05 by Welch’s *t*-test and an effect size ≥0.5 or ≤−0.5, whereas the gray dots indicate samples with *p* values >0.05 or effect size ranging from −0.5 to 0.5. The red, blue, and gray dots indicate increased, decreased, and unchanged relative abundance of bacterial groups, respectively. **e** and **f**, Relative and absolute abundance of *Bacillus*, respectively. I and II represent periods I and II. CK1 and CK2, tea plant monoculture in periods I and II; R1 and R2, intercropping with soybean in the tea garden in periods I and II. ^*^, *p* < 0.05; ^**^, *p* < 0.01.

Eight bacterial phyla (relative abundance >1%) were identified across all soil samples: Proteobacteria, Chloroflexi, Actinobacteria, Acidobacteria, Gemmatimonadetes, Bacteroidetes, Verrucomicrobia, and Patescibacteria (Fig. 3a). Compared with CK1, R1 treatment showed an increase in the relative abundance of Proteobacteria from 22.3% to 43.0% and decreases in Chloroflexi and Acidobacteria from 25.0% to 7.0% and 20.1% to 8.8%, respectively. In period II, intercropping enhanced the relative abundance of Proteobacteria and Acidobacteria from 18.2% to 23.6% and from 8.9% to 19.5%, respectively, and it decreased the relative abundance of Chloroflexi and Actinobacteria from 35.6% to 27.1% and from 23.6% to 12.9%. The dominant genera of each sample (top 15) were also assessed (Fig. 3b): AD3_norank, Gaiellales_norank, HSB OF53-F07, Acidobacteriales_norank, *Acidothermus*, subgroup 2, Gemmatimonadeceae_uncultured, Elsterales_norank, *Aeromonas*, Xanthobacteraceae_uncultured, *Gemmatimonas*, Saccharimonadales_norank, subgroup 6, *Bradyrhizobium*, and *Acidibacter*. Volcano plots were used to show the differences between pairs of soil samples (Fig. 3c and 3d). Compared with CK1, the R1 treatment significantly increased the relative abundance of Saccharimonadales_norank, Chitinophagaceae_uncultured, 67–14, Sphingomonadaceae, and rhizobia (*Allorhizobium*, *Neorhizobium*, *Pararhizobium*, and *Rhizobium*), and this was accompanied by lower relative abundance of Acidobacteriales_norank, Subgroups 2 and 13, Elsterales_norank, Candidatus Udaeobacter, and JG30-KF-AS9 (*p* < 0.05). Compared with CK2, the R2 treatment significantly enriched *Haliangium* and ADurb.Bin063–1 and decreased the relative abundance of FCPS473, Gaiellales_norank, and HSB OF53-F07.

Special attention was given to the taxonomy of *Bacillus* because of their ecological importance. The relative abundance of *Bacillus* in each sample was compared (Fig. 3e), and the results showed that intercropping with soybean decreased the relative abundance of *Bacillus* in both periods. As shown in Fig. 3f, intercropping decreased the absolute abundance of *Bacillus* in period I (*p* < 0.05). However, a different pattern was observed in period II: compared with monoculture, intercropping significantly increased the copy number of *Bacillus* in the tea garden (*p* < 0.05). These results were further supported by the total number of bacteria and spores detected using the traditional dilution-plate method (Supplementary Table 2).

### Isolation of *Bacillus* species and their effects on the growth and nodulation of legumes

**Figure 4:**
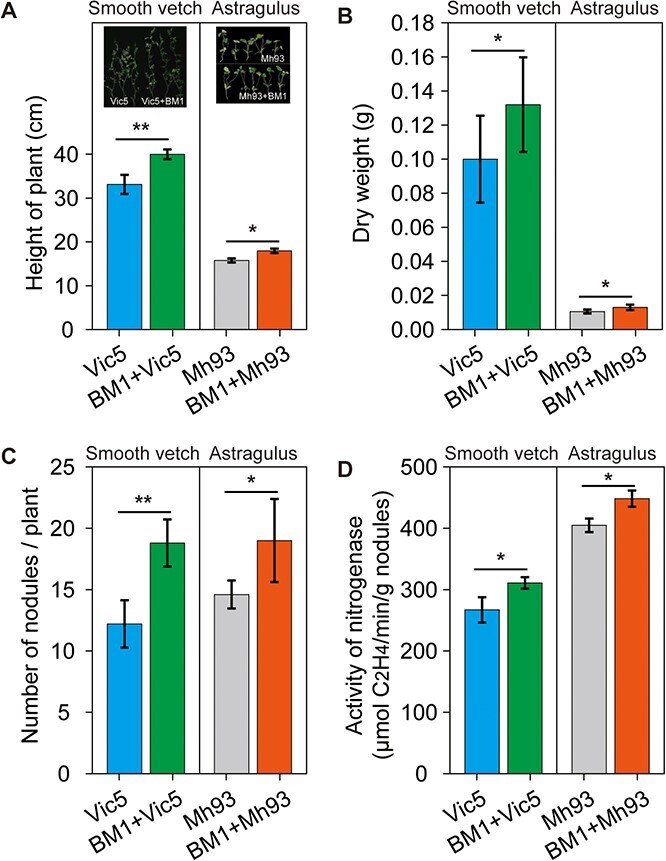
The effects of *B. amyloliquefaciens* BM1 on the performance of smooth vetch and *Astragulus* in pots. **a** Height of legumes. **b** Dry weight of legumes. **c** Number of nodules. **d** Nitrogenase activity. Vic5, *R. leguminosarum* Vic5; Mh93, *M. huakuii* Mh93. ^*^, *p* < 0.05; ^**^, *p* < 0.01.

Nine (B6, B8, B12, B19, B24, B42, B44, BM1, and BM10) and 7 (CB6, CM1, CM6, CM8, CM9, CM14, and CM15) *Bacillus* species were obtained from the CK2 and R2 soil samples, respectively. The symbiotic performance of each *Bacillus* species and *Bradyrhizobium diazoefficiens* USDA110 was assessed on soybeans (Supplementary Fig. 4). Among all *Bacillus* species, only strain BM1, identified as *Bacillus amyloliquefaciens*, produced an improvement in both nodule number and nitrogenase activity, thus exhibiting strong PGP properties. To confirm the effects of strain BM1 on the performance of cold-tolerant legumes (smooth vetch and *Astragulus sinicus* L*.)*, pot experiments were conducted in the laboratory (Fig. 4). Compared with single inoculation, co-inoculation with BM1 and Vic5 significantly increased smooth vetch height, dry weight, nodule number, and nitrogenase activity by 20.8%, 32.0%, 54.1%, and 16.5%, respectively. A similar trend was observed for co-inoculation with BM1 and Mh93 on *A. sinicus*, which increased the above parameters by 13.9%, 22.6%, 30.1%, and 10.8%, respectively.

### Strain BM1 improved the efficiency of the tea plant–SV intercropping system in winter

**Figure 5:**
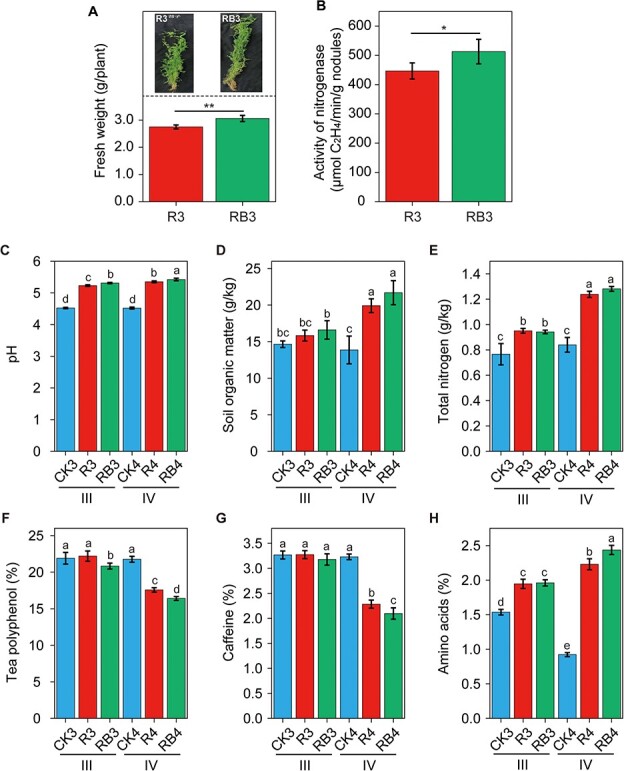
Effects of intercropping with smooth vetch on soil fertility and tea leaf secondary metabolites in field experiments. **a** Fresh weight of smooth vetch at 6 weeks post inoculation. **b** Activity of nitrogenase in single and dual inoculation. **c** Soil pH. **d** Soil organic matter. **e** Total nitrogen. **f** Tea polyphenols. **g** Caffeine. **h** Amino acids. Different letters represent significant differences (*p* < 0.05). III and IV represent periods III and IV in Fig. 1. CK3 and CK4, monoculture in periods III and IV; R3 and R4, intercropping with smooth vetch and inoculation with USDA110 in periods III and IV; RB3 and RB4, intercropping with smooth vetch and inoculation with USDA110 and BM1 in periods III and IV.

Based on the biomass of smooth vetch and *A. sinicus* (Fig. 4), smooth vetch was selected as an intercropping plant for the garden in winter. As shown in Fig. 5, compared with single inoculation, co-inoculation significantly increased the fresh weight of smooth vetch in the tea garden by 11.3% (Fig. 5a, *p* < 0.05). Similarly, the activity of nitrogenase was also increased by 14.8% in the co-inoculation treatment (Fig. 5b, *p* < 0.05). By contrast, planting of smooth vetch in the tea garden effectively enhanced soil pH values, regardless of inoculation mode and sampling time (Fig. 5c). Compared with CK3, the R3 and RB3 treatments did not affect the accumulation of SOM, although the R4 and RB4 treatments did so in period IV (Fig. 5d, *p* < 0.05). Compared with the corresponding CK, the R and RB treatments significantly increased TN levels by 23.4% (R3) and 22.1% (RB3) in period III and by 47.6% (R4) and 52.4% (RB4) in period IV. There was also a striking increase in TN between period III and period IV for the inoculated treatments (Fig. 5e, *p* < 0.05). In addition, intercropping systems had different effects on tea quality indices. Compared with the corresponding CK, the RB3 and RB4 treatments decreased the content of tea polyphenols in periods III and IV, with the minimum value obtained in period IV (Fig. 5f, *p* < 0.05). Intercropping did not affect the caffeine content in period III but decreased it in period IV (Fig. 5g, *p* < 0.05). By contrast, intercropping increased the content of amino acids in the leaves in both period III and IV, but particularly in period IV (Fig. 5h, *p* < 0.05). Similarly, the co-inoculation treatment produced a higher content of amino acids than the single inoculation treatment (*p* < 0.05).

Compared with CK4, single inoculation with *Rhizobium leguminosarum* Vic5 (R4 treatment) increased the total number of bacteria but not the spores or gene copies of *Bacillus* (Table 1). By contrast, co-inoculation with Vic5 and BM1 significantly increased all of the above parameters compared with all other treatments (*p* < 0.05). Accordingly, in period IV, 27, 27, and 24 *Bacillus* species were randomly isolated from the CK4, R4, and RB4 treatments, respectively (Supplemental Table 3), showing more kinds of *Bacillus* species in the RB4 treatment. Soybeans were meant to be planted in the same garden for the next intercropping cycle (soybean + smooth vetch), and the PGP effects of these strains on the performance of soybeans were therefore assessed when they were co-inoculated with Vic5. The results suggested that 6/27, 10/27, and 14/24 strains could efficiently improve both the growth of soybeans and the efficiency of symbiotic nitrogen fixation (Supplementary Fig. 5). Based on statistical analysis, the R4 treatment did not increase the ratio of PGP *Bacillus* compared with CK4, but the RB4 treatment did so significantly.

**Table 1:** Number of bacteria and spores in different treatments

Sample	Number of bacteria (×10^7^ CFU/g soil)	Number of spores (×10^5^ CFU/g soil)	Copies of *Bacillus* (×10^8^/g soil)
CK4	1.06 ± 0.22^c^	2.33 ± 0.58^b^	3.68 ± 0.22^b^
R4	2.17 ± 0.04^b^	4.00 ± 1.73^b^	3.05 ± 0.36^b^
RB4	2.96 ± 0.81^a^	16.67 ± 1.15^a^	10.60 ± 0.94^a^

Different letters indicate significant differences (*p* < 0.05). CK4, monoculture in period IV; R4, intercropping with smooth vetch and inoculation with USDA110 in period IV; RB4, intercropping with smooth vetch and inoculation with USDA110 and BM1 in period IV.

## Discussion

Intercropping is an important agricultural technique that has been used in China since ancient times. Previous studies have demonstrated that intercropped plants in tea gardens, such as Chinese chestnut, fruit, and soybean, are beneficial for improving soil nutrients and tea quality [4, 26]. In this study, legumes were selected as intercropping plants, demonstrating their potential for use in tea gardens. One important factor is that legumes will not compete with other plants for N because they can obtain the necessary N from the atmosphere through BNF. Another advantage is that legumes can be rapidly decomposed when they are turned into soil, increasing microbial counts and soil nutrients [16, 27]. This notion was previously confirmed by Farooq et al. [9], who examined peanut–tea intercropping and found that intercropping actively enhanced soil fertility and positively affected soil health. In this study, our data suggested that intercropping with soybean and smooth vetch could increase the soil TN content (Fig. 2c and Fig. 5e), consistent with previous studies. N is necessary for plant growth and amino acid synthesis. In a very recent study, Duan et al. [28] investigated the effects of soybean intercropping on the secondary metabolites of tea plants and found that intercropping particularly promoted amino acid synthesis when soybean was in the profuse flowering stage. This observation may partly support the finding of this study and help us to explain the physiological changes in the tea plants.

Our data also suggested that intercropping with either soybean or mung bean was helpful for increasing the level of SOM, particularly in period II (Fig. 2b and Supplementary Fig. 2b), suggesting that legume maturation in soils was extremely important for increasing soil fertility. It is worth noting that SOM is usually stable and changes slowly. However, compared with CK2, a significant increase in SOM was observed in the R2 treatment from 13.6 g/kg to 22.3 g/kg. This increase was probably attributable to a signal bias from legume residues in soil that were oxidized by K_2_Cr_2_O_7_-H_2_SO_4_ using our selected method, and the measured values of SOM were therefore greater than the true values. Thus, continuous application of intercropping in the same gardens should be maintained to obtain more information. In tea gardens, soil acidification can result in the accumulation of aluminum and a lack of phosphorus, potassium, and magnesium [6]. In this study, we found that soil pH increased (by approximately 0.9 units) after intercropping with smooth vetch, suggesting that this intercropping pattern was beneficial for the soil chemical environment. However, in a different study (currently unpublished), the data showed that soil pH decreased after intercropping. Therefore, a long-term intercropping experiment will be crucial for precisely simulating this phenomenon. In Nanjing, tea leaves are typically picked around China’s Tomb Sweeping Day (the 5th of April). Our data showed that after intercropping with soybean + smooth vetch, tea leaves in period IV had the highest quality during the tea-picking season, with the highest content of amino acids and the lowest contents of tea polyphenols and caffeine. Therefore, we concluded that long-term intercropping with legumes in tea gardens, as well as the application of beneficial *Bacillus* species, is a beneficial strategy for maintaining high soil fertility and tea quality.

High-throughput sequencing data revealed that Proteobacteria, Chloroflexi, Actinobacteria, and Acidobacteria were the top four phyla in the tea garden, consistent with the findings of Fu et al. [29] and Shen and Lin [16]. We also observed an obvious increase in the relative abundance of Proteobacteria in the flowering–podding period of soybean. It is well known that plants require sufficient N during the flowering period. Some studies have indicated that a wide range of N_2_-fixing bacteria belong to Proteobacteria [30, 31]. According to these results, we hypothesized that soybean utilized these special bacterial groups during the flowering period to help themselves obtain the necessary N. At the genus level, intercropping increased the relative abundance of rhizobia (*Allorhizobium*, *Neorhizobium*, *Pararhizobium*, and *Rhizobium*), which may plausibly support the above assumption. Compared with monoculture, intercropping with soybean significantly increased the relative abundance of *Haliangium* in period II (Fig. 3d). Previous studies have suggested that *Halingium* has PGP effects [32]. A recent study by Wang et al. [33] showed that legume straw (*Chamaecrista rotundifolia*) combined with chemical fertilizers and poultry manure increased the relative abundance of *Haliangium* in acidic tea soils, which accounted for 1.56% of the total bacteria. Hence, intercropping with soybean in tea gardens can promote specific bacterial populations. In period II, bacterial groups showed increasing and/or decreasing trends (Fig. 3d), but only a few could be identified to the genus or species level, which made it difficult to understand their ecological roles.

*Bacillus* species are widely distributed in agricultural soils and are associated with biological control of biotic diseases, as well as plant growth, quality improvement, and yield increase [34, 35]. In tea gardens, *Bacillus* species are frequently obtained from soil samples and root surfaces because of their acid-resistant and endogenous spore-forming characteristics [36]. In this study, our data suggested that both the relative and absolute abundances of *Bacillus* decreased in period I after intercropping with soybean, but an inconsistent trend was observed for period II, as relative abundance decreased but absolute abundance increased in the intercropping system. Although the relative abundance of microbial communities provides important insights into diversity and microbial community composition, it does not reflect the absolute abundance of each microbial group [37]. Previous investigations have reported both similar and opposite results with respect to relative and absolute abundance [24, 25, 38]. Thus, combining relative and absolute abundances can more accurately reflect the changes in microbial communities. According to these results, we hypothesized that organic amendments (soybean residues) in soils promoted the multiplication of *Bacillus*, which are involved in the decomposition of organic matter. This assumption was partially supported by Su et al. [39], who investigated the effects of long-term organic fertilization on the active soil bacterial communities using high-throughput sequencing and qPCR-based SmartChip assays. They found that organic fertilization dramatically enhanced the abundance of *Bacillus*. Similarly, Feng et al. [40] reported the effect of organic manure fertilization on agroecosystems, showing that *Bacillus asahii* responded to organic manure fertilization and became the dominant species after 2–4 years. In the present study, the increased absolute abundance of *Bacillus* in the R2 treatment suggested that intercropping with soybean could accelerate the soil carbon cycle in the tea garden, which in turn may have been beneficial to tea quality.

*Br*. *diazoefficiens* USDA110 is commonly recognized as a highly efficient N_2_-fixing strain when it forms symbiotic relationships with soybeans. Our data clearly demonstrated that co-inoculation of soybean with *Bacillus* species and USDA110 altered growth performance and significantly improved nodulation and BNF (Supplementary Fig. 4). These effects were also observed for combinations of BM1 and Mh93 on *A. sinicus* and BM1 and Vic5 on smooth vetch (Fig. 4). These data suggested that some plant growth-promoting rhizobacteria (PGPR) could positively interact with rhizobia and/or plants, improving growth performance. Similar findings were obtained by Korir et al. [20], who examined the co-inoculation effect of rhizobia and PGPB on common bean growth. They showed that co-inoculation of IITA-PAU987 and *Bacillus megaterium* significantly increased nodule weight and biomass compared with IITA-PAU alone in a low-phosphorus soil. A study by Tilak et al. [41] suggested that co-inoculation of pigeonpea with *Rhizobium* and either *Bacillus cereus*, *Pseudomonas putida* or *Pseudomonas fluorescens* contributed to a significant increase in plant growth, nodulation, and enzyme activity compared with single inoculation and uninoculated controls. Under saline-alkali conditions, Han et al. [42] obtained 278 *Bacillus* species from soils and found that *B. cereus* could promote the growth of sinorhizobia and alleviate the effects of saline-alkali stress on nodulation, suggesting a key role for *Bacillus* species in shaping rhizobia–host interactions in soybean. These results suggested that co-inoculation with rhizobia and PGPR could improve the growth performance of legumes and confirmed their applicability in agriculture. In this study, among all *Bacillus* species, *B. amyloliquefaciens* BM1 was considered to be the most efficient strain for improving plant performance based on growth, nodulation, and nitrogenase activity, which was consistent with previous studies. This strain was previously reported as a PGPR that enhanced soybean nodulation when co-inoculated with *Bradyrhizobium japonicum*, and this improvement was ascribed to the production of auxin, gibberellins, and salicylic acid [43]. In a recent study, Sibponkrung et al. [44] identified synergistic effects between *Bacillus velezensis* S141 and *Br*. *diazoefficiens* USDA110 on nodule growth and N_2_ fixation, revealing that IAA and cytokinin produced by S141 promote USDA110, thus producing larger nodules. In addition to phytohormone production, Rajendran et al. [45] suggested that *Bacillus* strains NR4 and NR6 could mediate the growth of *Rhizobium* sp. IC3123 and increase nodule number through siderophore production. In our study, strain BM1 showed synergistic effects with rhizobial strains (USDA110, Mh93, and Vic5) and/or legumes, which may have been due to the production of unknown metabolites. In addition, our data also suggested that intercropping with both BM1 and USDA110 led to an increase in the ratio of PGP *Bacillus*, which was in turn beneficial to the sustainable development of tea gardens. However, the interactions among PGPR, rhizobia, and legumes are extremely complex, and the process may be affected by other factors, such as soil nutrient status, soil microorganisms, and/or some unknown conditions. Therefore, further investigations are needed to expand our understanding of this field.

## Conclusion

In summary, we investigated the effects of legumes as intercropping plants in a tea garden and confirmed that the “soybean in summer and smooth vetch in winter” strategy was beneficial for increasing soil fertility and improving tea quality. In such intercropping systems, PGP *Bacillus* species can act as biostimulants and inocula to improve the growth performance of legumes and tea plants.

## Materials and methods

### Tea–legume intercropping in a field experiment

The field experiment was conducted at Jiangsu Bocha Tea Industry Co. Ltd., Nanjing, China. The location has a northern semitropical monsoon climate with an average annual temperature, rainfall, and frost-free period of 15.7°C, 1072.9 mm, and 224 days. The examined tea garden has been subjected to monoculture management for approximately 20 years. A randomized complete block design with two treatments was used for the field experiments. Each treatment contained three rows of tea plants (each row: 15 m × 3 m = 45 m^2^). Monoculture was used as the control (CK), and soybean was planted as the intercropping plant. For inoculation, bacterial inoculum was prepared according to the following process. *Br. diazoefficiens* USDA110 was cultured using tryptone yeast medium (TY) at 28°C for 5 days (180 rpm). Bacterial cells were obtained by centrifugation (6000 g for 10 min), re-suspended and precipitated five times in sterile distilled water, and ultimately preserved in ddH_2_O. Bacterial density was diluted to approximately 10^8^ cells/mL. Before sowing, *Br*. *diazoefficiens* USDA110 inoculum was applied to the seed in the form of a slurry. Slurry enriched with bacterial communities and soil was thoroughly mixed according to the following ratio: 200 g seeds, 200 g soil, 10 mL bacterial suspension, and 40 mL water. Soybean (*Glycine max*) of the “Hefeng47” variety was sown in late May 2019 (45 kg/ha) and had flowered and/or podded in early June 2019; this period was defined as period I. The soybeans were then turned into the soil until late August 2019 (for approximately 40 days), and this period was defined as period II. The conceptual model associated with the experimental design and the sampling periods is shown in Fig. 1. In the monoculture treatment, the weeds were cut aperiodically, and their residues were turned into the soil. In the intercropping rows, no weeds or only a few weeds were present, as they were almost or completely controlled by the legumes. All other management practices were the same between monoculture and intercropping. Soil and tea leaf samples were collected from the two cover patterns in period I (CK1 and R1) and period II (CK2 and R2). At the same time, mung bean of the “Sulv4” variety was also used as an intercropping plant in the tea garden following the method described above.

### Plant performance and soil analysis

The height of each plant in the pot experiment was recorded using a ruler. The nodules on each legume plant were counted manually, and the nitrogenase activity was determined using an acetylene reduction assay according to Ning et al. [46]. Catechin and caffeine contents were analyzed using HPLC (Waters, USA) according to national standards (GB/T8313–2008), and amino acid content was determined according to the ninhydrin colorimetry method (GB/T8314–2013). Soil samples were collected from the junctional zones (5–15 cm) of the roots of tea plants and soybeans (period I) and roots of tea plants and legume residues (period II). Obvious plant roots and residues were carefully removed. Soil samples were immediately transported to the laboratory. Each soil sample was divided into three parts. One part was stored at −80°C for extraction of bacterial DNA and RNA, the second part was stored at 4°C for cell counting and microbial screening, and the third part was air-dried in the laboratory for soil chemical analysis. Soil pH was measured using a pH meter (PHS-3CT) in a 1:2.5 soil/water mixture. SOM was analyzed in 0.5 g soil using 10 mL of 0.136 mol/L K_2_Cr_2_O_7_-H_2_SO_4_ with 3–4 drops of phenanthroline indicator, and the mixture was titrated with 0.2 mol/L standard FeSO_4_ solution [47]. TN was measured for 0.5 g soil with 2 g accelerator (K_2_SO_4_: CuSO_4_:Se (w:w:w) = 100:10:1) and 5 mL of H_2_SO_4_ (overnight). Soil samples were heated using a boiling furnace (200°C for 15 min, 380°C for 3–4 h) until the color turned off-white, and incubation was then maintained for another 1 h. The liquid was diluted with ddH_2_O to a volume of 100 mL. A continuous flow analytical system (San^++^ System, Skalar, Holland) was used to measure TN.

### Validation of the nitrogen metabolism-related genes *CsGS* and *CsGOGAT*

Total RNA was extracted from tea leaves using the RNAsimple Total RNA kit (Tiangen, Beijing, China). cDNA was then synthesized using the PrimeScript RT reagent kit (TaKaRa, Tokyo, Japan) according to the manufacturer’s protocol. The primers of *CsGs* were F: 5′-GCC AAT CCC AAC AAA TAA GAG G-3′ and R: 5′-TAT CCG CAC CAA TAC CAC AG-3′, and the primers of *CsGOGAT* were F: 5′-CGA AAA ACG GTG ACA GAT G-3′ and R: 5′-AGG AAG AGC GAC GAG AAT G-3′. qPCR was performed in a 20-μL reaction mixture that contained 80–100 ng cDNA, 200 nM of each primer, and 10 μL LightCycler 480 SYBRGREEN I Master Mix (Roche, Basel, Switzerland). All reactions were performed in duplicate in 96-well plates. Real-time PCR was performed under the following conditions: 95°C for 30 s, and 40 cycles at 95°C for 10 s and 60°C for 30 s. Following melting curve analysis at 95°C for 15 s, 60°C for 60 s, and 95°C for 15 s, *β*-actin expression was used as the internal control. Finally, the expression levels in each sample were analyzed using the 2^−ΔΔCt^ method.

### DNA extraction, PCR amplification, and sequencing

DNA was extracted from soil samples (approximately 0.5 g) using the FastDNA SPIN Kit for Soil (MP Biomedicals) following the manufacturer’s instructions. The primer set 338F 5′-ACT CCT ACG GGA GGC AGC AG-3′ and 806R 5′-GGA CTA CHV GGG TWT CTA AT-3′ with barcode was used to amplify bacterial V3-V4 hypervariable regions of the 16S rRNA gene. PCR amplification was performed according to Huang et al. [48]. After DNA purification (E.Z.N.A. Cycle Pure Kit, OMEGA) and quantification (NanoDrop 2000), PCR products were sent to Biozeron Bio-Technology Co., Ltd. (Shanghai, China), and sequencing was performed on the Illumina MiSeq platform.

### Isolation of *Bacillus* spp. from tea–soybean intercropping soil

*Bacillus* species were isolated from soil samples according to the following procedure. In brief, 10 g soil was added to an Erlenmeyer flask that contained 90 ml sterilized water. The flask was then successively shaken (180 rpm) for 20 min and heated in a water bath (80°C) for 15 min. Soil suspensions were serially diluted in a 10-fold gradient (10^−2^, 10^−3^, and 10^−4^). Then, 0.1 mL of each soil suspension was inoculated onto minimal medium (MM) containing the following (per L): mannitol (10 g), yeast powder (0.5 g), K_2_HPO_4_ (0.5 g), MgSO_4_ (0.097 g), and NaCl (0.1 g). The plates were placed in an incubator for 2–3 days (30°C), and then colonies were randomly selected from the plates. The strains were purified by streaking on fresh medium 2–3 times and identified based on their 16S rRNA gene sequences.

### Quantification of *Bacillus* by qPCR

qPCR was conducted using a Roche Light Cycler 480 system. The primer set BacF/R1378 [49] was used for the quantification of *Bacillus* with SYBRGREEN-based reactions (three replications). The 20-μL reaction mixture contained 0.25 μL template DNA, 200 nM of each primer, and 10 μL LightCycler 480 SYBRGREEN I Master Mix (Roche, Basel, Switzerland). Reactions were run for 5 min at 95°C and 45 cycles of 15 s at 95°C, 30 s at 60°C, and 30 s at 72°C, followed by plate reading. Melting curve analysis and electrophoresis were used to check the amplification specificity. A qPCR standard was generated using plasmid DNA from one clone containing each of the above genes. A series of 10-fold dilutions of the standard template were used per assay. The R^2^ value for the standard curve was 0.99, and the qPCR efficiency was ≥0.98 for the quantitative assays.

### The effects of *Bacillus* spp. on nodulation and N_2_ fixation of legumes

*Br*. *diazoefficiens* USDA110 was cultured as described above. *Bacillus* spp. were cultured in tryptic soy broth and washed accordingly. Each *Bacillus* strain population was then adjusted to approximately 10^8^ CFU/ml. For the nodulation assays, sterilized soybeans were planted in glass tubes (parceled with black papers) containing Fahraeus medium and grown under controlled conditions (16 h light/8 h darkness, 30°C). Cells of USDA110 (1 mL) were inoculated onto the roots of soybean seedlings when their roots reached approximately 2–3 cm. For co-inoculation, a liquid volume (1 mL) that contained an equal amount of USDA 110 and each of the *Bacillus* strain solutions was added to the roots of the soybean seedlings. N-free Hoagland’s solution was used to supply the necessary elements. After 4 weeks, the growth of soybean seedlings was recorded, and nodulation and the associated nitrogenase were analyzed accordingly. Using the same method, the effects of co-inoculation of the most efficient *Bacillus* with *R. leguminosarum* Vic5 on smooth vetch or with *Mesorhizobium huakuii* Mh93 on *A. sinicus* were confirmed after 6 weeks.

### Strain BM1 enhanced the performance of tea–smooth vetch intercropping

To enhance the effects of intercropping in the tea garden, smooth vetch was selected as an intercropping legume because of its cold resistance and greater biomass yield during the winter. In this experiment, all treatments were performed in the same field area. The soybean rows planted in summer were divided into two parts, which were used for single and dual inoculations (Fig. 1). Therefore, there were three treatments overall: monoculture, intercropping smooth vetch with Vic5 only, and intercropping smooth vetch with co-inoculation of Vic5 and BM1. The seedling handling, bacterial suspension, and inoculation method were performed as described above. The seeds of smooth vetch were sown in mid-Oct 2020 (45 kg/ha), and the smooth vetch was turned into the soil in early Apr 2021 (flowering–podding period, period III) for approximately 30 days (early May 2021, period IV). Soil and tea leaf samples were collected in periods III and IV. Soil properties, plant growth parameters, total amino acid content, tea polyphenols and caffeine in tea leaves, nodulation, and nitrogenase activity were all determined as described above.

### Data analysis

The data obtained by Illumina sequencing were analyzed according to QIIME [50]. In brief, raw sequences were separated based on their unique barcodes, and the barcode and primer sequences were then removed. The reads were clustered into OTUs based on 97% sequence similarity. Finally, a representative sequence for each OTU was assigned to sequences deposited in the SILVA database (v132). The relative abundance of each bacterial group and alpha diversity indices, including OTU number, Chao 1, and Shannon, were analyzed with QIIME according to the tutorial. STAMP (version v2.13) was used to analyze differences in the relative abundances of bacterial groups between each pair of samples [51]. Nonmetric multidimensional scaling (NMDS) was performed to explore the differences in bacterial communities based on Bray–Curtis distances using the vegan R package. PERMANOVA was performed to determine whether the bacterial communities showed significant differences among soil samples (vegan R package). One-way ANOVA and Fisher’s least significant difference (LSD) post hoc test (Bonferroni method) were used to determine the differences among soil samples using R (version 4.0), and differences between sample pairs were confirmed using *t*-test analysis.

## Supplementary Material

Web_Material_uhac046

## Data Availability

The authors declare that all data in this study are available within the paper and its supplementary files. The raw sequence data reported in the paper have been deposited in the Genome Sequence Archive (https://bigd.big.ac.cn/gsa/) under accession number CRA005114.
