# Supplementary material for: Tea plant–legume intercropping simultaneously improves soil fertility and tea quality by changing Bacillus species composition
Source: Hortic Res. 2022 Feb 19;9:uhac046. doi: 10.1093/hr/uhac046 (PMC9123240; doi:10.1093/hr/uhac046)
Supplement: Web_Material_uhac046 [file web_material_uhac046.docx]

***Horticulture Research***

**Supplementary Data**

**Title: The plant-legume intercropping simultaneously improves soil fertility and tea quality by changing *Bacillus* species composition**

**Authors:** Zhi Huang^1^, Chunhong Cui^2^, Yajun Cao^1^, Jinghui Dai^1^, Xiaoyue Cheng^1^, Shaowei Hua^1^, Wentao Wang^1^, Yu Duan^3^, Hui Wang^1^, Lixiang Zhou^2^, Wanping Fang^3^, Zengtao Zhong^1^

^1^College of Life Sciences, Nanjing Agricultural University, Nanjing 210095, China

^2^College of Resource and Environment Sciences, Nanjing Agricultural University, Nanjing 210095, China

^3^College of Horticulture, Nanjing Agricultural University, Nanjing 210095, China

Correspondences: Wanping Fang (fangwp@njau.edu.cn) and Zengtao Zhong ([ztzhong@njau.edu.cn](mailto:ztzhong@njau.edu.cn))

**Supplementary Table 1** Alpha diversity indices in intercropping and monoculture soils

|  | OTUs | Shannon | Chao 1 |
| --- | --- | --- | --- |
| CK1 | 3420.3±874.5^a^ | 6.78±0.54^a^ | 4843.8±1313.3^a^ |
| R1 | 2902.3±319.5^a^ | 6.76±0.35^a^ | 3684.9±319.9^a^ |
| CK2 | 2503.3±281.5^a^ | 6.25±0.23^a^ | 3201.2±537.6^a^ |
| R2 | 2361.3±158.2^a^ | 6.45±0.16^a^ | 3098.5±161.9^a^ |

Different letters present significant differences (*p* <0.05).

**Supplementary Table 2** Total number of bacteria and spores in CK2 and R2 treatments

| Sample | Number of bacteria  (×10^7^ CFU/g soil) | Number of spores  (×10^5^ CFU/g soil) |
| --- | --- | --- |
| CK2 | 2.50±0.01^b^ | 1.00±0.01^b^ |
| R2 | 26.60±9.70^a^ | 40.00±2.20^a^ |

Different letters represent significant differences (*p* < 0.05).

**Supplementary Table 3** Number of each *Bacillus* species isolated from different treatments

|  | CK4 | R4 | RB4 |
| --- | --- | --- | --- |
| *Bacillus velezensis* | 2 | 8 | 3 |
| *Bacillus cereus* | 0 | 0 | 2 |
| *Bacillus sonorensis* | 10 | 13 | 1 |
| *Bacillus zanthoxyli* | 3 | 1 | 4 |
| *Bacillus ferrooxidans* | 0 | 0 | 1 |
| *Bacillus acidiceler* | 0 | 0 | 2 |
| *Bacillus pseudomycoides* | 0 | 0 | 1 |
| *Bacillus safensis* | 0 | 0 | 4 |
| *Bacillus aerius* | 0 | 0 | 1 |
| *Bacillus manliponensis* | 5 | 0 | 1 |
| *Bacillus siamensis* | 0 | 1 | 0 |
| *Bacillus proteolyticus* | 0 | 2 | 0 |
| *Bacillus altitudini* | 0 | 1 | 0 |
| *Bacillus drentensis* | 0 | 1 | 0 |
| *Bacillus thuringiensis* | 2 | 0 | 0 |
| *Bacillus albus* | 2 | 0 | 0 |
| *Bacillus toyonensis* | 2 | 0 | 0 |
| *Bacillus wiedmannii* | 1 | 0 | 0 |
| *Bacillus amyloliquefaciens* | 0 | 0 | 4 |
| Total | 27 | 27 | 24 |

CK4, monoculture; R4, intercropping with smooth vetch and inoculation with USDA110; RB4, intercropping with smooth vetch and inoculation with USDA110 and BM1.


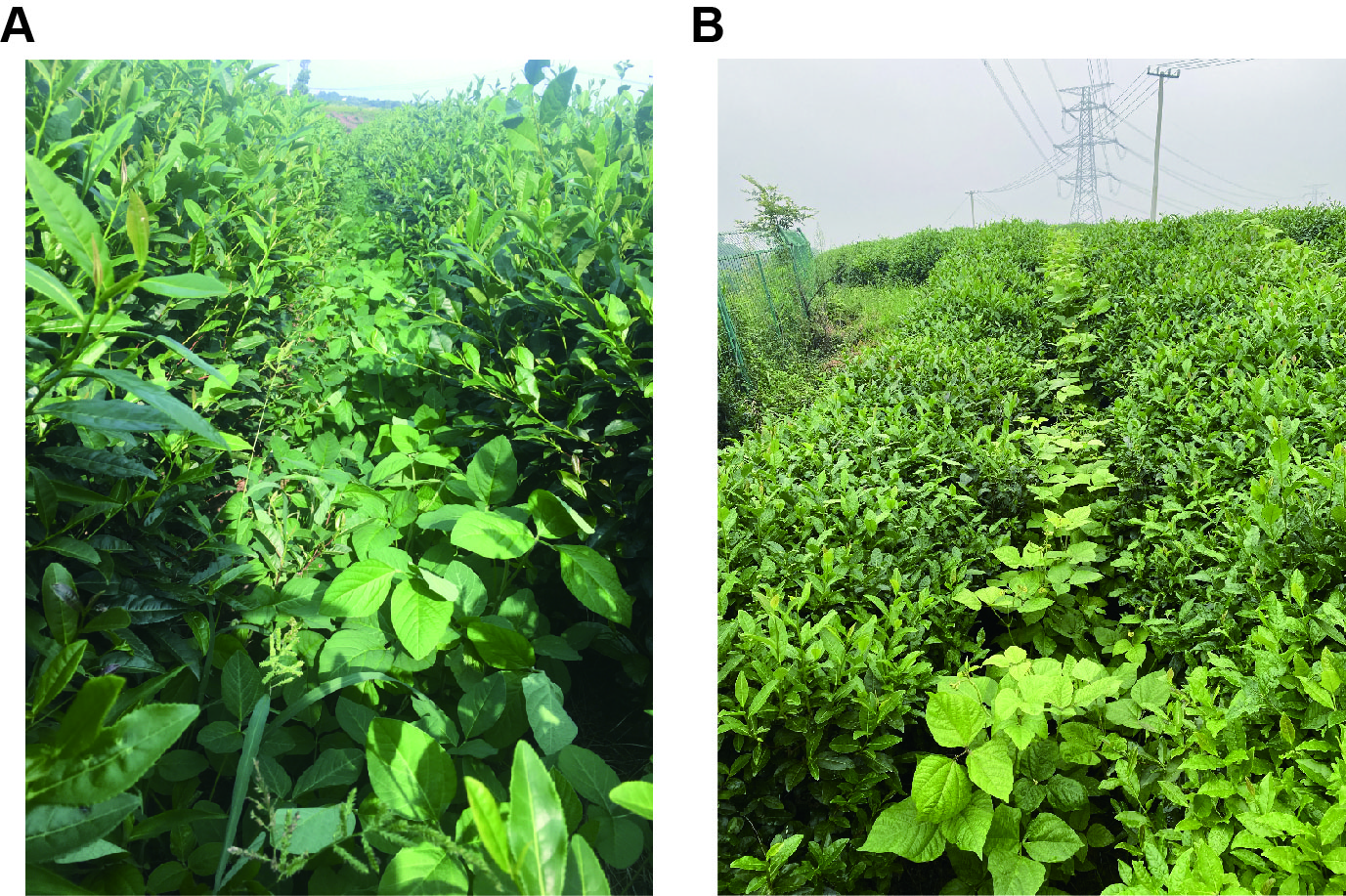


**Supplementary Fig. 1** The performance of soybean and mung bean in the tea garden. **a**, soybean; **b**, mung bean.


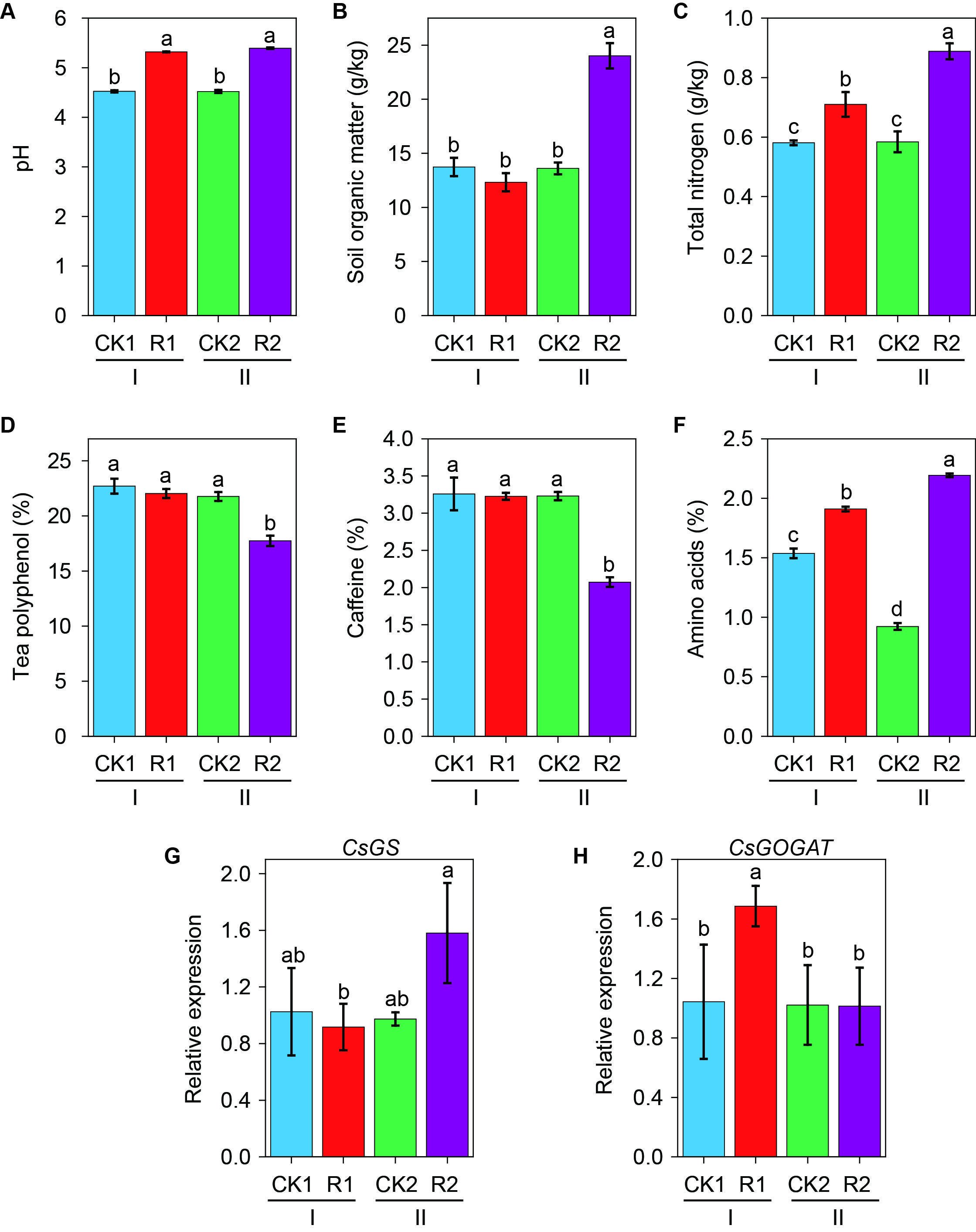


**Supplementary Fig. 2** The effects of monoculture and intercropping (mung bean-tea plant) under field conditions. **a** soil pH. **b** soil organic matter. **c** total nitrogen. **d** tea polyphenol in tea leaves. **e** caffeine in tea leaves. **f** amino acids in tea leaves. **g** the relative expression of *CsGS*. **h** the relative expression of *CsGOGAT*. CK1 and CK2, monoculture of tea plant in period I and period II, respectively; R1 and R2, intercropping with soybean in the tea garden in period I and period II, respectively. All data were presented as mean ± SD (n=3). Different letters represent significant differences (*p* < 0.05).


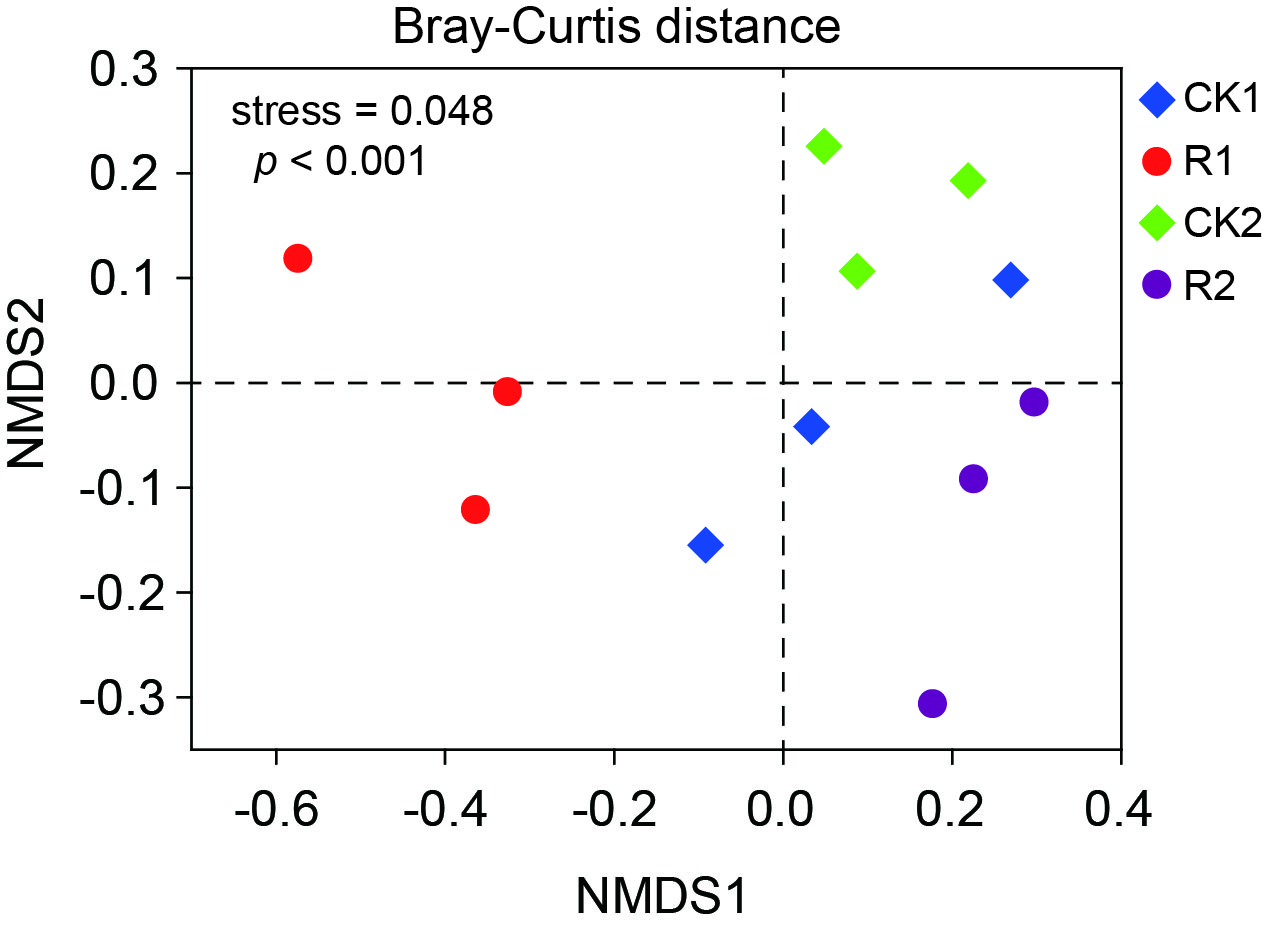


**Supplementary Fig. 3** Nonmetric multidimensional scaling (NMDS) plots of bacterial communities based on the Bray-Curtis distance. CK1 and CK2, monoculture of tea plant in period I and period II, respectively; R1 and R2, intercropping with soybean in the tea garden in period I and period II, respectively.


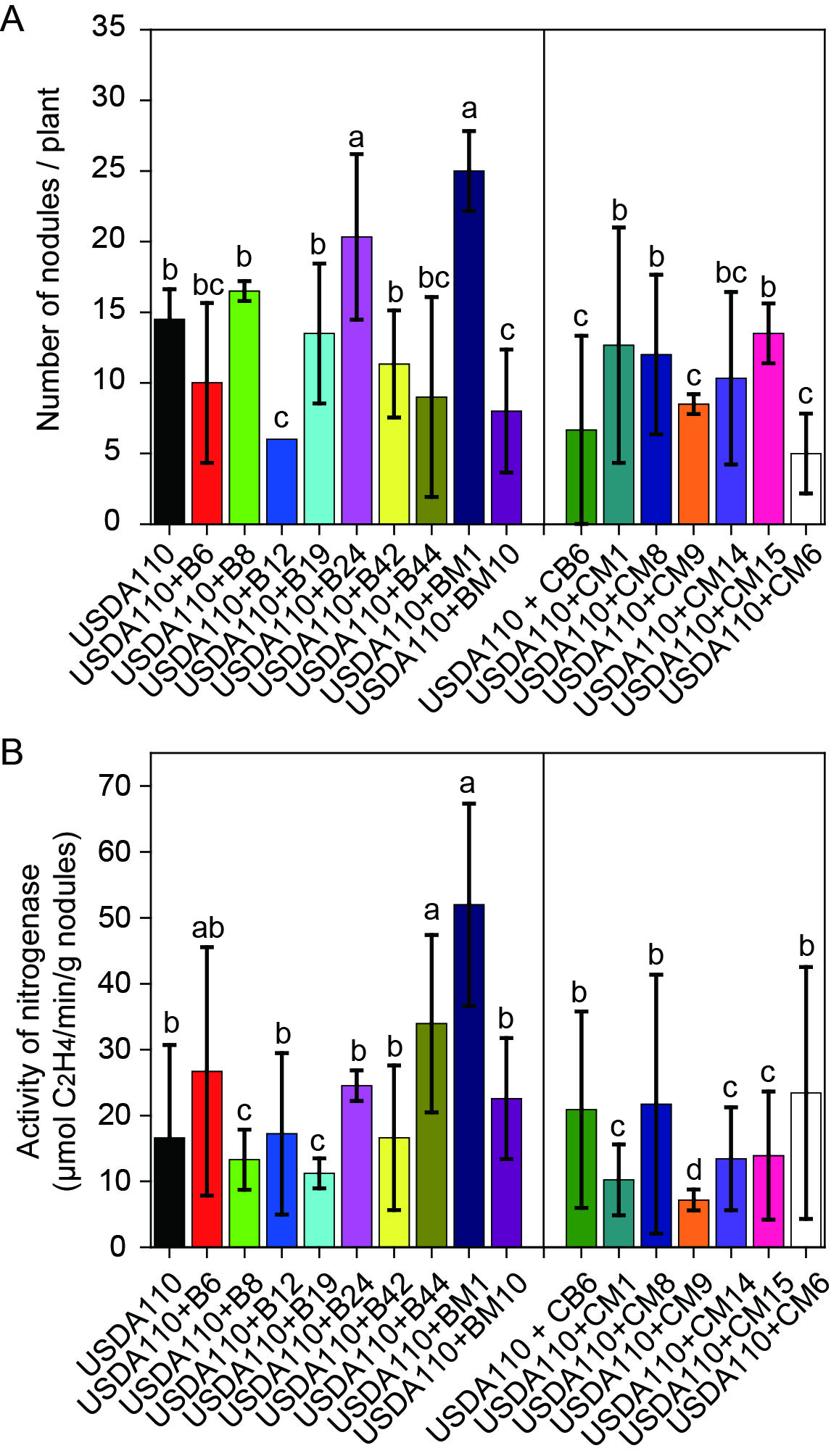


**Supplementary Fig. 4** Symbiotic performance of *Bradyrhizobium diazoefficiens* USDA110 and *Bacillus* species on soybean (*Glycine max*)*.* **a** Number of root nodules on soybean; **b** Nitrogen-fixation activities of root nodules were measured by acetylene reduction assay. Error bars show the standard deviation based on three replicates. Different letters present significant difference (*p* < 0.05)


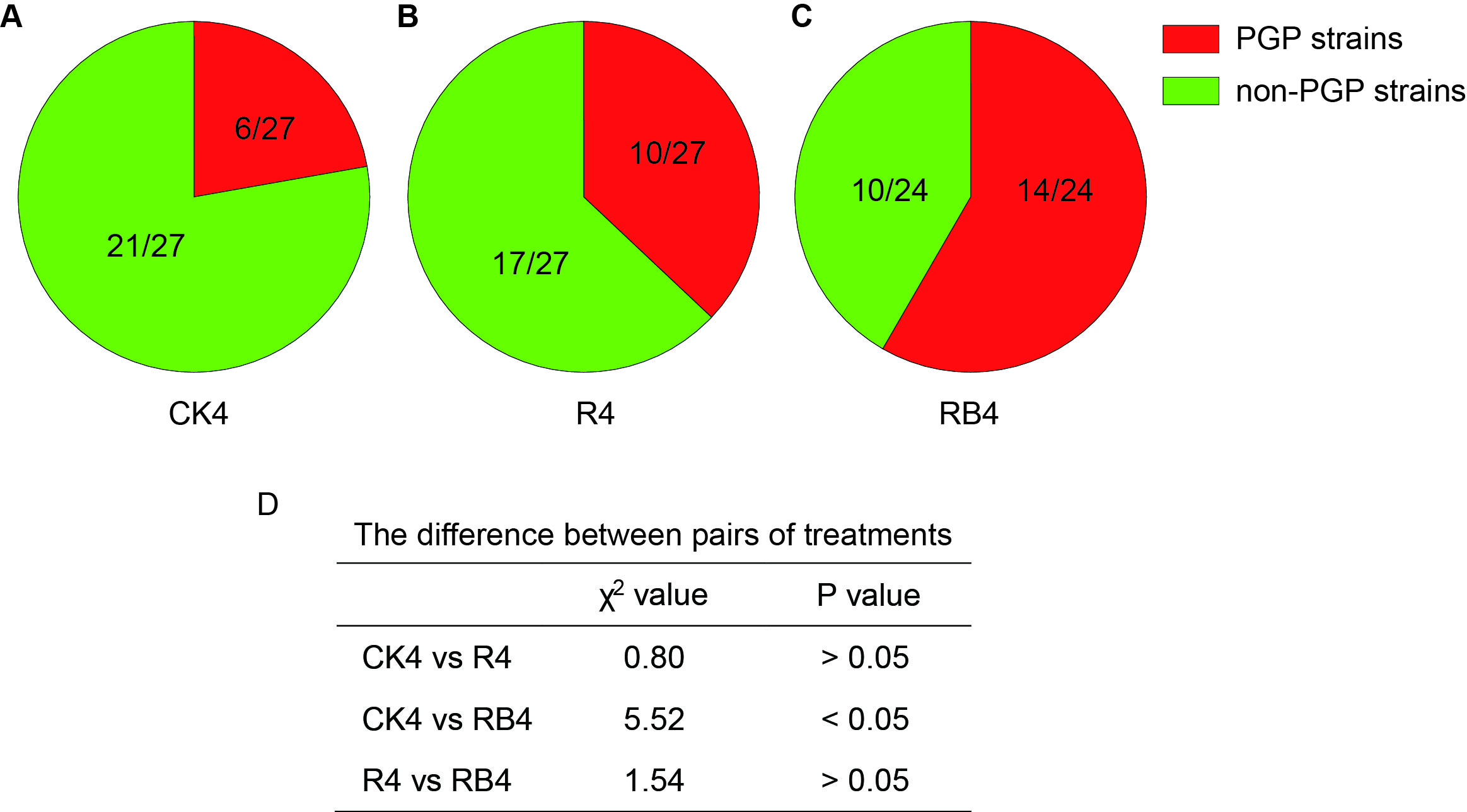


**Supplementary Fig. 5** Number of plant growth-promoting *Bacillus* species in CK4, R4 and RB4 treatment (**a**, **b** and **c**, respectively), and the statistical analysis of the ratios of PGP *Bacillus* (**d**).
